# In Vitro Interactions of Dietary Fibre Enriched Food Ingredients with Primary and Secondary Bile Acids

**DOI:** 10.3390/nu11061424

**Published:** 2019-06-25

**Authors:** Susanne Naumann, Ute Schweiggert-Weisz, Julia Eglmeier, Dirk Haller, Peter Eisner

**Affiliations:** 1 ZIEL-Institute for Food & Health, TUM School of Life Sciences Weihenstephan, Technical University of Munich, 85354 Freising, Germany; dirk.haller@tum.de (D.H.); peter.eisner@ivv.fraunhofer.de (P.E.); 2 Fraunhofer Institute for Process Engineering and Packaging (IVV), 85354 Freising, Germany; ute.weisz@ivv.fraunhofer.de (U.S.-W.); julia.eglmeier@ivv.fraunhofer.de (J.E.); 3 Chair of Nutrition and Immunology, TUM School of Life Sciences Weihenstephan, Technical University of Munich, 85354 Freising, Germany

**Keywords:** bile acid binding, bile acid excretion, cholesterol, colorectal cancer, in vitro digestion, critical micelle concentration

## Abstract

Dietary fibres are reported to interact with bile acids, preventing their reabsorption and promoting their excretion into the colon. We used a method based on in vitro digestion, dialysis, and kinetic analysis to investigate how dietary fibre enriched food ingredients affect the release of primary and secondary bile acids as related to viscosity and adsorption. As the main bile acids abundant in humans interactions with glyco- and tauroconjugated cholic acid, chenodesoxycholic acid and desoxycholic acid were analysed. Viscous interactions were detected for apple, barley, citrus, lupin, pea, and potato derived ingredients, which slowed the bile acid release rate by up to 80%. Adsorptive interactions of up to 4.7 μmol/100 mg DM were significant in barley, oat, lupin, and maize preparations. As adsorption directly correlated to the hydrophobicity of the bile acids the hypothesis of a hydrophobic linkage between bile acids and dietary fibre is supported. Delayed diffusion in viscous fibre matrices was further associated with the micellar properties of the bile acids. As our results indicate changes in the bile acid pool size and composition due to interactions with dietary fibre rich ingredients, the presented method and results could add to recent fields of bile acid research.

## 1. Introduction

Primary bile acids are synthesized in the liver from cholesterol and stored in the gall bladder. The most common types of primary bile acids are cholic acid (CA) and chenodesoxycholic acid (CDCA), which are conjugated either with taurine or glycine to form water-soluble bile salts [1]. After a meal gall bladder contraction is stimulated and bile salts are secreted. In the intestinal tract, they act as detergents facilitating the absorption of dietary lipids and fat-soluble vitamins [2].

Bile acids are reabsorbed by active transport and passive diffusion and transported back to the liver by the hepatic portal vein. The bile acid pool, which contains about 2.5 to 5 g of bile acids, is thereby recycled 4 to 12 times a day. This process, called the enterohepatic circulation, is highly efficient and recycles about 95% of the bile acid pool. Daily, 400 to 800 mg of bile acids escape this circulation and becomes substrate for microbial transformation to secondary bile acids [3].

The most common secondary bile acids are desoxycholic acid (DCA) and lithocholic acid. These result from deconjugation and dehydroxylation of primary bile acids by the human gut microbiota [4]. As lithocholic acid is the most hydrophobic bile acid, reabsorption rates back into the enterohepatic circulation are small [5]. DCA, on the other hand, is less hydrophobic and is reabsorbed in the colon, transported back to the liver and is recycled with CA and CDCA. Therefore, the bile acid pool consists of about 40% CA, 40% CDCA, 20% DCA and trace amounts of lithocholic acid [2].

Besides lipid metabolism bile acids are involved in several physiological processes. The hepatic production of primary bile acids represents the main pathway to remove an excess of cholesterol from the body [6]. Recent studies indicate that bile acid pool size and bile acid composition contribute to the regulation of gut microbial community structures [7]. Additionally, bile acids support glucose regulation and energy homeostasis, are involved in several cellular signalling pathways and are ligands for numerous nuclear hormone receptors [5].

In contrast, bile acids are also associated with a number of disease phenotypes. High concentrations of secondary bile acids promote carcinogenesis in the colon [8]. Changes in bile constituents are linked to the development of biliary stones [9]. Cytotoxicity due to microbial changes of bile acid structures may cause inflammation, apoptosis, and cell death. Changes in the size or composition of the bile acid pool are further related to cardiac dysfunctions, liver diseases and diabetes [2,5].

Dietary fibres are reported to interact with bile acids preventing their reabsorption and promoting their transit to the colon. Reducing the reabsorption rate of bile acids affects the bile acid pool size, increases the hepatic synthesis of primary bile acids from cholesterol, and thus, changes the composition of the bile acid pool [10]. Using a pig model Gunness et al. demonstrated that a decrease in blood cholesterol in the presence of oat β-glucan was associated with restricted bile acid diffusion, reduction of circulating bile acids and a change in bile acid profile [11]. Furthermore, a change in the bile acid profile towards a higher concentration of 12α-hydroxylated bile acids was associated with insulin resistance in humans [12]. Interestingly, various types of dietary fibres or fibres with different physio-chemical characteristics were related to changes in bile acid profiles of rats [13]. Dietary fibre intake is thus directly related to bile acid metabolism, its various physiological functions and the development of bile acid related diseases. A further understanding of dietary fibre - bile acid interactions is thus needed to understand fibre related changes of bile acid profiles as a measure of physiological homeostasis [14].

Food processing following the industrial and agricultural revolutions entailed the development of a “Western diet” high in calories, sugars and animal fats and low in dietary fibre. Therefore, daily consumption is far below recommendations for fibre intakes, which range from 25 to 35 g/day depending on country specific guidelines [15]. Dietary fibres are characterized by their resistance to enzymatic degradation within the upper gastrointestinal tract. However, they differ greatly in their structural and nutritional properties [15,16]. Elucidation of the structures and properties of dietary fibres responsible for their health promoting effects is thus needed to make specific recommendations and promote the fibre fortification of food.

Hypotheses to explain the interaction of dietary fibres with bile acids can be ascribed to two main effects: Dietary fibres directly adsorb bile acids (e.g. by hydrophobic interactions) and a viscous matrix formed by the dietary fibres reduces bile acid release rates, respectively [10].

Due to bacterial alterations of bile acids and dietary fibres in the colon and the structural diversity of bile acids in animals, in vivo studies to directly measure interactions between dietary fibres and human bile acids are very limited. Yet, common in vitro methods to measure the interaction of dietary fibres with bile acids lacked in precision and differed significantly from the physiological conditions in the human body. A recent comparison of in vitro methods in our laboratory revealed that the combination of in vitro digestion, dialysis and kinetic analysis enables the differentiation of adsorptive and viscous interactions [17]. In the present study, this method has been extended to the analysis of the main components abundant in human bile.

The aim of this study is to reveal viscous and adsorptive interactions of dietary fibre enriched food ingredients on conjugated primary bile acids (CA and CDCA) and DCA as an important representative of the secondary ones. Furthermore, the dependence of conjugation and the degree of bile acid hydroxylation should be examined. By this means, we want to contribute to a further understanding of the health promoting effects of dietary fibres as related to bile acid metabolism.

## 2. Materials and Methods

### 2.1. Bile Acids

**Figure 1:**
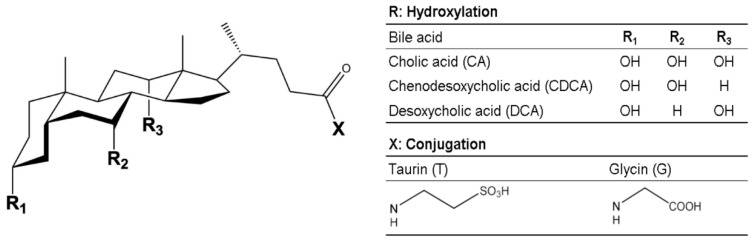
Chemical structure of primary and secondary bile acids mainly abundant in human bile.

Taurocholic acid sodium salt hydrate (CAS 345909-26-4), sodium taurochenodeoxycholate (CAS 6009-98-9), sodium glycocholate hydrate (CAS 338950-81-5), sodium glycochenodeoxycholate (CAS 16564-43-5), sodium taurodeoxycholate hydrate (CAS 207737-97-1), and sodium glycodeoxycholate (CAS 16409-34-0) were purchased from Merck KGaA (Darmstadt, Germany). Structural differences in hydroxylation and conjugation of the investigated primary and secondary bile acids are given in Figure 1.

### 2.2. Chemicals and Enzyme Preparations

L-α-lecithin (Egg Yolk, Highly Purified-CAS 8002-43-5), α-amylase from human saliva (Type IX-A, 1000–3000 units/mg protein), pancreatin from porcine pancreas (8 × USP specifications) and pepsin from porcine gastric mucosa (3,200-4,500 units/mg protein) were purchased from Merck KGaA (Darmstadt, Germany). All other reagents and chemicals were of analytical grade and supplied by VWR (Darmstadt, Germany).

### 2.3. Dietary Fibre Enriched Food Ingredients

A resistant starch preparation NUTRIOSE^®^ FM 06 was provided by Roquette Frères (Lesrem, France). A maize bran derived preparation SOFABRAN 184-80 was purchased from Limagrain Céréales Ingrédients (Saint-Ignat, France). An oat preparation VITACEL HF 401-30 (extracted from oat spelt bran), a potato preparation VITACEL KF 200 and a wheat preparation VITACEL WF 600 were supplied by J. Rettenmaier & Soehne GmbH & Co. KG. An apple preparation Herbacel AQ Plus Apple—A 09 and a citrus preparation Herbacel AQ Plus Citrus - N (derived from apple or citrus fruit pomace) were provided by Herbafood Ingredients GmbH (Werder, Germany). A barley preparation SANACEL^®^ betaG was obtained from CCF GmbH & Co. KG (Gehren, Germany). A pea preparation Emfibre EF 200 (derived from yellow pea cotyledons) was supplied by Emsland-Stärke GmbH (Emlichheim, Germany). A lupin preparation was obtained from lupin cotyledons processed on a pilot scale plant based on the method described by D’Agostina et al. [18].

### 2.4. Dietary Fibre Composition

Soluble and insoluble dietary fibre contents were determined by enzymatic–gravimetric analysis according to AOAC 991.43 [19].

### 2.5. Viscosity

Viscosity measurements were conducted using a rotational rheometer (Physica MCR 301, Anton Paar, Graz, Austria). Triple determinations were performed using a parallel plate geometry (diameter: 25 mm, shear gap: 1 mm) (PP25-SN23060, Anton Paar, Graz, Austria) at constant temperature of 37 ± 0.1 °C. An aliquot of 2 g of the in vitro digested fibre preparation was positioned in the centre of the tempered plate geometry, the opposite plate was lowered and the sample was trimmed prior to measurement. The samples were pre-sheared at a shear rate of 5 s^−1^ for 20 s and allowed to rest for 20 s before starting the measurement. The viscosity η was monitored as a function of the shear rate, which was increased in a logarithmic scale ranging from 0.01–1000 s^−1^.

### 2.6. In Vitro Interactions with Bile Acids

#### 2.6.1. In Vitro Digestion

The in vitro digestion was performed according to the harmonized protocol, developed by the COST INFOGEST network, with slight modifications [20]. The digestion procedure included an oral phase, a gastric phase and an intestinal phase. The dietary fibre enriched food ingredients were weighed in Erlenmeyer flasks and diluted with demineralized water to obtain a final dry matter concentration of 50% (*w/w*). Simulated digestion fluids (simulated salivary fluid, simulated gastric fluid and simulated intestinal fluid) were exactly prepared as published in Minekus et al. [20]. To avoid microbial growth, 0.04% of sodium azide was added to all electrolyte fluids to reach a concentration of 0.02% in the final digestion mixture. The diluted samples were mixed with pre-warmed (37 °C) simulated salivary fluid (ratio 50:50 (*w/w*)) containing α-amylase to achieve 75 IU·mL^−1^ in the final mixture to start the oral phase. The mixture was incubated at 37 °C for 2 min and then subjected to gastric phase digestion by adjusting the pH of the mixture to 3.0 with 1 M HCl and mixing with simulated gastric fluid (ratio 50:50 (w/w)) containing pepsin and lecithin to reach a final concentration of 2000 IU·mL^−1^ and 0.17 mM in the digestion mixture. After incubation for 2 h, the intestinal digestion phase was started by raising the pH to 7.0 with 1 M NaOH and adding simulated intestinal fluid (ratio 50:50 *w/w*), which contained pancreatin and bile acids to reach 100 IU·mL^−1^ of trypsin activity and 10 mM in the digestion mixture. Due to overlapping retention times in HPLC-DAD (Section 2.6.3), the interactions of dietary fibre enriched ingredients were examined separately for primary and secondary bile acids. To study interactions with primary bile acids, a mixture (1:1:1:1) containing taurocholic acid (TCA), glycocholic acid (GCA), taurochenodesoxycholic acid (TCDCA) and glycochenodesoxycholic acid (GCDCA) was used. To investigate interactions with secondary bile acids, taurodesoxycholic acid (TDCA) and glycodesoxycholic acid (GDCA) were selected as representatives and were used in equal proportions. The mixture was incubated for 2 h. The digestion was performed in a shaking water bath at 37 °C. A blank digestion was performed by using a mixture of simulated digestion fluids and the same concentration of lecithin, pepsin, pancreatin and bile but the sample was replaced with demineralized water.

#### 2.6.2. Dialysis and kinetic analysis

In total, 4 g of the in vitro digested samples, with 10 mM primary or secondary bile acid mixtures, was transferred into membranes prepared from 16 mm Servapor^®^ 12–14 kDa cut-off dialysis tubings (SERVA Electrophoresis GmbH, Heidelberg, Germany). Dialysis was carried out against 36 mL of 50 mM phosphate buffer (pH 7) containing 0.02% sodium azide using a shaking water bath at 150 rpm and 37 °C. 100 μL-aliquots of dialysate were taken at 1, 2, 4, 8, 12, 24 and 48 h dialysis time and analysed for permeated bile acids by HPLC (Agilent, Santa Clara, CA, USA).

Bile acid release kinetics were assessed using SigmaPlot^®^, version 12.5 (Systat Software Inc., San Jose, CA, USA). To describe the diffusion under dialysis the triplicate measurements at seven dialysis times were fitted using non-linear regression. According to the first-order kinetic described in Equation (1), the concentration of bile acids after reaching equilibrium (Cf) and the apparent permeability rate constant (k) were calculated [21,22].

(1) $$C_{t}=C_{f}\times \;\left(1-{e\;}^{-\;k\;t}\right)$$

Bile acid adsorption was calculated with respect to the dry matter concentration in the in vitro digesta (μmol/ 100 mg DM).

#### 2.6.3. Bile Acid Quantification Using HPLC

Bile acids were analysed using an Agilent HPLC series 1200 system (Agilent, Santa Clara, CA, USA) equipped with Open Lab CDS software version 2.3, a G1379 degasser, a G1312B binary gradient pump, a G1367D thermo autosampler, a G1316B column oven, and a G1315C diode array detector. The column used was an Ascentis^®^ Express C18 (150 × 4.6 mm i.d.; 2.7 μm particle size) from Supelco^®^ Analytical (Merck KGaA, Darmstadt, Germany) operated at 40 °C. The binary gradient consisted of a water solution containing 0.012% formic acid and 5 mM ammonium acetate (solvent A) and methanol containing 0.012% formic acid and 5 mM ammonium acetate (solvent B) [23,24]. A binary gradient program was applied as follows: Linearly gradient 70% to 100% B in 10 min; held at 100% B for 5 min followed by re-equilibration at 70% B for 5 min. The injection volume was 10 μL and column flow was 0.6 mL/min. Bile acids were detected at 200 nm and quantitated using peak area analysis.

### 2.7. Statistical Analysis

Results are reported as mean ± standard deviation. Significance is shown by a *p* ≤ 0.05. The results were assessed using R version 3.2.4 (https://www.R-project.org) [25]. Homogeneity of variance and normal distribution were tested by Bartlett and Shapiro–Wilk test. Two-way ANOVA and one-way ANOVA with post-hoc Tukey test were performed to separate significant means.

## 3. Results

### 3.1. Dietary Fibre Composition of Food Ingredients

Dietary fibre compositions of fibre enriched food ingredients derived from different sources are given in Table 1.

The ingredients used in this study differed in the total dietary fibre contents (TDF) and in the ratio of water-soluble and insoluble fibre components. While TDF was highest for wheat (98.5 g/100 mg DM), lowest TDF was detected in the barley derived ingredient (29.2 g/100 mg DM). However, despite fully soluble resistant starch preparation, the barley derived ingredient showed the highest proportion of soluble fibre (67.3%). High soluble proportions were also found in apple (17.6%), potato (16.5%) and citrus (15.7%) derived ingredients [17], whereas the fibre enriched food ingredients derived from oat, maize, wheat and pea were mainly composed of insoluble fibre.

### 3.2. Viscosity of in Vitro Digested Dietary Fibre Enriched Food Ingredients

**Figure 2:**
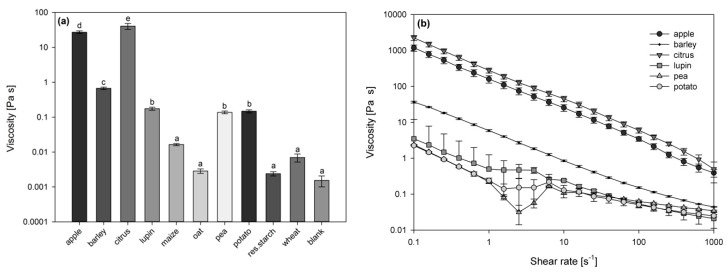
Comparison of viscosity at shear rate 15 s^−1^ (**a**) and viscosity as a function of the shear rate (**b**) of in vitro digested dietary fibre enriched food ingredients derived from different sources. Different letters indicate significant differences on a *p* ≤ 0.05 level basis (*n* = 3).

Since only small shearing forces occur in the gastrointestinal tract, the viscosity of in vitro digested dietary fibre enriched food ingredients were compared at a shear rate of 15 s^−1^ (Figure 2a) [17,21]. The oat, wheat, and resistant starch preparations did not significantly increase the viscosity of the digesta, as the viscosity was comparable to the blank digestion. The chymes containing apple and citrus fibre preparations were highly viscous in comparison to all other analysed samples. Viscous networks were also formed by barley, lupin, pea and potato derived preparations, which significantly increased the viscosity in comparison to the blank digestion.

Due to low values of viscosity, no definite shear rate dependency was observed for in vitro digesta of oat, wheat and resistant starch preparations. All other samples showed a similar pattern of shear thinning at high shear rates (Figure 2b). This flow behaviour is typical of entangled polymer solutions. By increasing the shear rate the polymers align with the direction of the shear as well as with the direction of the shear gradient. Thus, they partially disentangle, which decreases the flow resistance.

### 3.3. Interactions with Bile Acids

**Figure 3:**
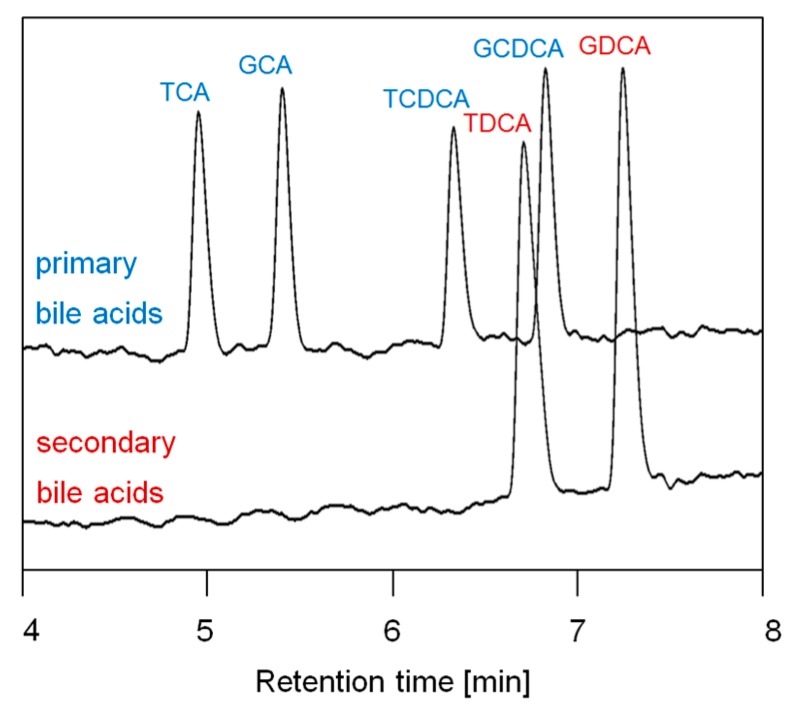
Separation of primary and secondary bile acids using HPLC-DAD at 200 nm (glycocholic acid (GCA), taurocholic acid (TCA), glycochenodesoxycholic acid (GCDCA), taurochenodesoxycholic acid (TCDCA), glycodesoxycholic acid (GDCA), taurodesoxycholic acid (TDCA)).

Complete separation was achieved for separate analysis of primary and secondary bile acids by HPLC-DAD (Figure 3). Bile acid concentrations and bile acid release kinetics were calculated. Correlation coefficients for non-linear regression of bile acid release were in the range of 0.963 to 0.999, which shows the high agreement of the kinetic fitting with the experimental data.

**Figure 4:**
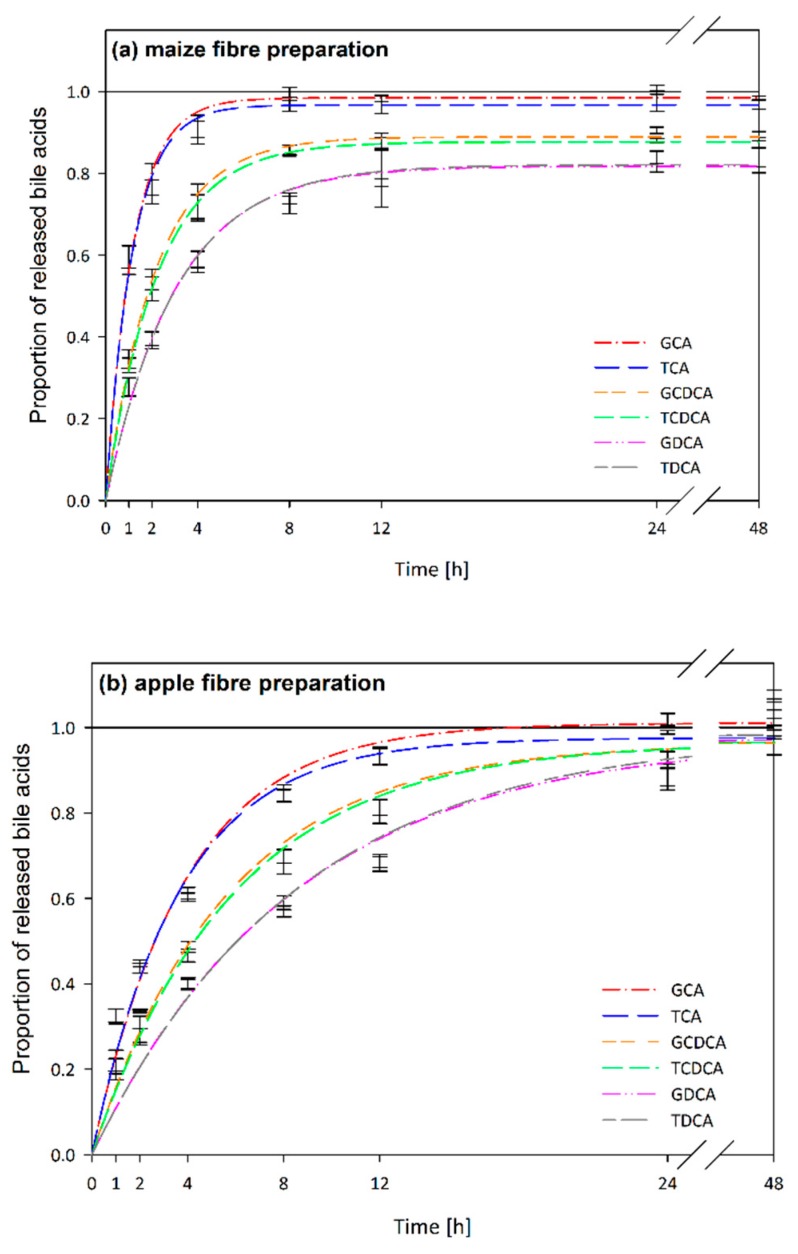

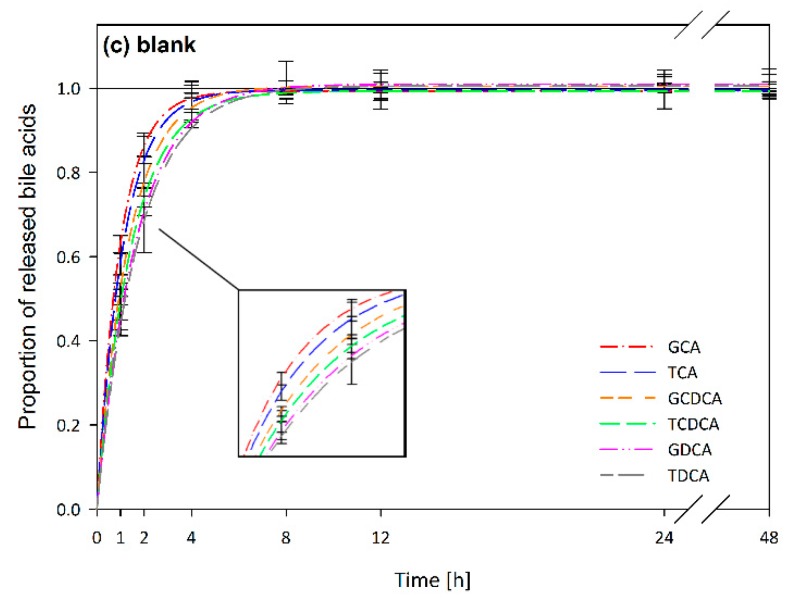
Diffusion kinetics of bile acid release (glycocholic acid (GCA), taurocholic acid (TCA), glycochenodesoxycholic acid (GCDCA), taurochenodesoxycholic acid (TCDCA), glycodesoxycholic acid (GDCA), taurodesoxycholic acid (TDCA)) of in vitro digested (**a**) maize fibre preparation, (**b**) apple fibre preparation and (**c**) blank digestion.

In Figure 4, the diffusion kinetics of bile acid release are exemplified by maize fibre preparation (a) showing adsorptive effects and by apple fibre preparation (b) showing viscous effects in comparison to blank digestion (c).

#### 3.3.1. Adsorptive Effects as Related to Bile Acid Structures

Significant adsorption in comparison to the blank digestion was detected for barley, oat, lupin and maize fibre enriched food ingredients (Table 2). Adsorptive effects depended strongly on the structure of the bile acids as shown for the maize preparation in Figure 4a. Dihydroxy bile acids (CDCA and DCA) were adsorbed to a higher degree than trihydroxy bile acids (CA), with DCA showing highest adsorption (two-way ANOVA, *p* < 0.001). Conjugation had no significant effect on the adsorption (two-way ANOVA, *p* = 0.6) of bile acids. Therefore, the data for glyco- and tauroconjugated CA, CDCA and DCA are summarized in Table 2.

#### 3.3.2. Adsorptive Effects as Related to Dietary Fibre Sources

CA were adsorbed by barley (1.19 ± 0.13 µmol/100 mg DM) and oat preparations (1.07 ± 0.11 µmol/100 mg DM). Adsorption of dihydroxy bile acids (CDCA and DCA) was higher and significant (in comparison to the blank digestion) in barley, oat, lupin and maize preparations. In maize (Figure 4a) and barley derived ingredients, the adsorption of DCA was significantly higher than for CDCA. Highest adsorption rates for all analysed bile acids were found for the ingredients derived from barley and oat. Adsorption of DCA was significantly higher in barley derived preparation than in all other analysed samples.

#### 3.3.3. Viscous Effects as Related to Bile Acid Structures

Blank digestion revealed that bile acid release in aqueous media varied depending on bile acid structures (Figure 4c). This was more evident in samples, which increased the viscosity of the digesta e.g., apple preparation (Figure 4b). Release rates differed depending on the conjugation and the degree of hydroxylation of the bile acids. Slightly reduced release rates were observed for tauroconjugated bile acids in comparison to glycoconjugated bile acids in the blank digestion and in the samples (Figure 4b,c), Table 3). Two-way ANOVA revealed significant differences in apparent permeability rates for glyco- and tauroconjugated bile acids with *p* = 0.003. Hydroxylation affected the release rates to a greater extent: Apparent permeability rates were higher for trihydroxycholic acids (CA) than dihydroxycholic acids (CDCA and DCA) with DCA showing the lowest release rates (two-way ANOVA, *p* < 0.001).

#### 3.3.4. Viscous Effects as Related to Dietary Fibre Sources

For samples showing no adsorption, the apparent permeability rate (k) correlates with the viscosity of the fibres [17]. Accordingly, a wheat fibre and a resistant starch preparation did not interact with the bile acids. Kinetic analysis revealed interactions for all other analysed samples by means of retarded bile acid permeability rates (Table 3). The highest retardation of release of all bile acids was found in highly viscous chymes containing apple (Figure 4b) and citrus fibre preparations. Pea and oat preparations showed slight reductions of CDCA and DCA release rates, while the apparent release rate constant for CA was not significantly different from the blank digestion.

As only minor adsorption rates of CA were detected, viscous interactions of adsorbing ingredients (barley, oat, maize, lupin) were compared based on the apparent permeability rate constants of CA: While oat and maize preparations did not show significant viscous interactions, apparent permeability rate was slowed down by 65% by barley and by 48% by lupin derived ingredients in comparison to the blank digestion.

## 4. Discussion

Our results indicate that both viscous and adsorptive interactions of dietary fibres with bile acids are influenced by the properties of the fibre ingredients as well as by bile acid structure.

### 4.1. Adsorptive Interactions as Related to Bile Acid Structures

The degree of bile acid hydroxylation strongly influenced adsorptive interactions with dietary fibre enriched food ingredients: Dihydroxycholic acids were adsorbed more strongly (DCA > CDCA) than trihydroxycholic acids (CA). CA were adsorbed only to a minor extent by barley and oat preparations. CDCA and DCA showed larger adsorption rates of up to 26%. These were significant in barley, oat, lupin and maize preparations. The results of our study support the hypothesis of a hydrophobic linkage between bile acids and dietary fibre components. This was already proposed by several authors mostly focusing on differences between dihydroxy-bile acids and trihydoxy-bile acids [27,28,29,30]. Cornfine et al. further described bile acid binding to be positively correlated with the degree of fibre acetylation [31]. However, the exact mechanism remains to be fully elucidated. As soluble dietary fibres (like pectin, arabinoxylans and mixed linkage β-glucans) are hydrophilic, our results suggest that soluble fibre components do not contribute to the adsorptive capacity of the dietary fibre enriched ingredients. Accordingly, no direct molecular interaction with bile acids could be established for β-glucan, the soluble fraction of oat, by Gunness and Gidley [32]. Adsorption could thus be related to insoluble dietary fibre structures [33]. Furthermore, contributions of components associated to the dietary fibres could contribute to the adsorption, which was already proposed for lignin, proteins and phytochemical structures [34,35,36].

Hydrophobicity of bile salts decreases in the order of DCA > CDCA > CA, which correlates with the results of our study describing a significant decrease in adsorption following this order [5]. However, although glycine-conjugates are more hydrophobic than tauro-conjugates, no significant influence resulting from conjugation was detectable. This is in line with the recent finding of Parker et al. focusing on the adsorption–desorption behaviour of bile salts to hydrophobic surfaces [37]. The structure function relationships of bile salts were studied using dual polarisation interferometry and atomic force microscopy. The authors described that under physiological conditions simulating the small intestinal milieu (pH 7) glycine as well as taurine are fully charged increasing the solubility of the bile acids in a similar way. The minor role of the conjugation is further substantiated by flexible attachment by a four carbon chain separating the conjugating group from the main amphiphilic group. This explains why minor structural changes in bile acid hydroxylation have a much greater impact on adsorption than the major structural differences resulting from changes in the conjugated group [37]. The concordance of our results with this study focusing on adsorption to hydrophobic surfaces further corroborates the hypothesis of a hydrophobic linkage.

### 4.2. Viscous Interactions as Related to Bile Acid Structures

The conjugation of bile acids slightly affected the apparent release rate with tauroconjugated bile acids being released more slowly than glycoconjugated bile acids. Hydroxylation had a greater impact on apparent release rate; effects were significant in the blank digestion (Figure 4c) and enhanced in increasingly viscous matrices e.g., formed by apple fibre (Figure 4b). Release rates decreased with increasing hydrophobicity (DCA < CDCA < CA) [5]. We propose these findings to be mainly associated with the micellar properties of the bile acids, which are driven by hydrophobic effects and hydrogen bonding [38]. Referring to the equation of Stokes and Einstein (2), the diffusion (D) at constant temperature (T) and viscosity (η) is influenced exclusively by the radius (r) of the diffusing particles (k_B_: Boltzmann’s constant) [39].

(2) $$D=\frac{k_{B\;}T}{6\;\pi \;\eta \;r}$$

In the case of bile acids, this radius depends on the proportion of micelles in the solution and the number of molecules within a micelle (aggregation number (N_agg_)). In solution, the concentration of monomeric bile acids increases with increasing concentration until the critical micellar concentration (CMC) is reached and the monomer concentration saturates [38]. As recommended by Minekus et al., a total bile acid concentration of 10 mM was used in our study [20]. During dialysis, this concentration dilutes to 1 mM. Since critical micelle formation concentrations are between 0.9 and 18 mM, this results in different micellar proportions for the different bile acids. Differences in the aggregation number represent an additional parameter influencing the micelle diameters (Table 4).

According to Table 4 highest CMC and lowest aggregation numbers are described for CA, which therefore form less and smaller micelles causing faster diffusion compared to CDCA and DCA. Analogously, a decrease of diffusion rates of CDCA > DCA is expected by the combination of CMC and the aggregation number (Table 4).

The data displayed in Table 4 refers to aqueous solutions, in which bile salts self-assembly to primary and secondary micelles due to hydrophobic effects and hydrogen bonding [38]. Under physiological conditions bile salts form mixed micelles with phospholipids. This increases the capacity of the bile salts to solubilize cholesterol and lipids. These mixed micelles are greatly expanded, show higher aggregation numbers and lower CMC in comparison to micelles formed in aqueous solutions [40]. A recent study has shown that the internal structure of mixed micelles is independent of the lipid concentration [41]. During the in vitro digestion applied in our study bile salts were mixed with phospholipids. We therefore suggest that the micelles, formed under these in vitro conditions, resemble the mixed micelles in the intestine under physiological conditions. Reabsorption of mixed micelles due to micellar diffusion is greatly retarded [40]. Therefore, it is assumed that the in vivo reabsorption of bile salts can be explained by the uptake of monomeric constituents that are in equilibrium with the micellar structures [42]. These reabsorption conditions are represented by the in vitro system, as the dialysis membrane (cut-off: 12–14 kDa) prevents micelles from diffusion. In addition to a slower diffusion of the micelles to the membrane interface, the decrease of the apparent permeability rates CA > CDCA > DCA, can be explained by the higher concentration of monomeric bile acids as controlled by the CMC of the individual bile acids (Table 4). Micellization is further controlled by the conjugation of the bile acids: Tauroconjugated bile acids show slightly lower CMC values than glycoconjugated bile acids [43], which explains the slightly reduced apparent permeability rates observed for tauroconjugated bile acids in comparison to glycoconjugated bile acids.

### 4.3. Bile Acid Interactions as Related to Dietary Fibre Sources

The source and the structural composition of the tested dietary fibre enriched food ingredients highly affected the degree of interactions with bile acids. While some ingredients showed no significant or only minor interaction (wheat, resistant starch, pea, and potato), others showed predominantly viscous (apple, citrus) or adsorptive interactions (oat). A combination of viscous and adsorptive interactions was observed for barley, lupin and maize derived ingredients.

In comparison to the blank digestion, no consistent change of bile acid release was observed for the wheat fibre preparation. This contradicts the in vitro results obtained by centrifugation and described by Kahlon et al., who reported bile acid binding of 30.4% on equal TDF basis considering cholestyramine as 100% bound [44]. However, our results correspond to an in vivo study conducted by Bosaeus et al., who did not find an effect of wheat bran supplementation on bile acid excretion in ileostomy patients [45].

Consistent with our results, dextrin based resistant starches (RS4), like NUTRIOSE^®^FM, are known as a non-viscous fibre [46,47]. Increased faecal excretion of bile acids was described for native granular starch (RS2), retrograded, crystalline, nongranular starch (RS3), and chemically modified or re-polymerized starch (RS4) [48,49,50,51]. Contradictory data is described by Chezem et al., who did not observe a significant increase of faecal bile acid excretion after RS2 in rats [52]. However, they reported highest bile acid excretion after a starch-rich diet, which is in line with the in vitro data supplied by Simsek and El [49]. In addition to a possible influence of digestible starch on bile acid excretion, RS-induced changes in microbiota metabolites may alter bile acid absorption and thus influence faecal bile acid excretion [48,53]. It therefore remains to be clarified to what extent bile acid excretion in faeces can be attributed to adsorptive interactions between RS and bile acids. In our study dextrin-based RS did not show adsorptive interactions with bile acids. As RS encompass a wide variety of structures, further studies are needed to test the transferability of our results to other types of RS.

We found only minimal interactions of pea fibre with CDCA and DCA. Although pea has shown to lower serum cholesterol levels in vivo, further studies do not indicate that cholesterol lowering is due to interactions with bile acids [54,55,56]. The cholesterol lowering effect, however, is assumed to be attributed to pea protein or inhibition of cholesterol synthesis by short chain fatty acids because of colonic fermentation of the pea fibre [54,57]. Our results support these hypotheses as they do not indicate any direct influence of pea fibre on bile acid metabolism.

Potato fibre also interacted minimally with bile acids, which was shown by a slight but significant retardation of the bile acid release for CDCA and DCA. Therefore, we hypothesize that potential health effects of potato fibre are not directly related to interactions with bile acids. This is supported by the findings of Lærke et al., who reported no significant reduction of plasma cholesterol or glycaemic response after potato fibre intervention in rats [58].

Apple fibre preparation formed highly viscous chymes, which significantly slowed down bile acid release by up to 80% while no adsorptive interactions were detected. The viscous properties may be attributed to its high soluble fibre content of 15.7 g/ 100 mg DM. Accordingly, Parolini et al. described activation of CYP7A1, the rate limiting enzyme of bile acid synthesis after intervention with highly viscous apple pectin. This was ascribed to an increase in faecal loss of bile acids, which was also described in two studies conducted by Sembries et al. [57,59,60]. Our results suggested that this influence on bile acid metabolism may be attributed to the formation of a viscous chyme matrix reducing bile acid diffusion and absorption. A similar flow behaviour and bile acid interaction as in apple was observed in citrus fibre enriched ingredient. This is in line with the study conducted by Bosaeus et al., who found a significant increase in ileostomy bile acid excretion after citrus pectin [45]. Besides pectin, insoluble fibre components may interact with bile acids by formation of entangled fibre networks [17,61].

The cholesterol-lowering effect of some oat and barley-based food products is already described and mostly attributed to the viscosity increase of the digesta induced by β-glucan. Accordingly, EFSA allows health claims for foods enriched in β-glucan derived from both barley and oat although the exact mechanism responsible for cholesterol reduction is still not elucidated [62,63]. However, it is already known that the cholesterol-lowering effect is strongly influenced by the dose and processing as related to the refinement and molecular weight of the β-glucan enriched products [64]. Recent studies also indicate that, in addition to viscosity effects, β-glucan may decrease the permeability of the intestinal mucus [65]. Since the oat fibre used in our study showed only a minor proportion of water-soluble dietary fibre (1.5%), including β-glucan, the influence of β-glucan on the investigated bile acid interactions is probably negligible. This is also confirmed by the measurement of shear viscosity, which resulted in a viscosity of 0.003 Pa s (shear rate 15 s^−1^, Figure 2a), which did not differ significantly from the value observed after blank digestion. Adsorptive interactions must therefore be caused by insoluble dietary fibre or other components of the food matrix. This is in line with the study performed by Zacherl et al., who investigated the bile acid binding capacity of heat damaged oat fibre. Although the viscosity of the fibre was almost completely lost due to the thermal treatment, a dose-dependent bile acid binding of up to 26% was observed. This binding capacity was suggested to result from hydrophobic interactions [66]. In our former study we already hypothesized that the adsorption of the barley product was independent of its β-glucan content (19 ± 2%). However, the described interactions with TCA could nonetheless mostly be ascribed to the viscosity-increasing properties of the water-soluble β-glucan-rich fraction [17]. The current study has been extended to the analysis of six different bile acids, showing DCA to be adsorbed almost four times more than the previously investigated CA. The adsorption shown in both oat and barley derived ingredients could help to explain the cholesterol lowering effects already found in extracts and fractions low in β-glucan as well as in low molecular weight β-glucan hydrolysates [33,64,67,68,69]. Further studies are needed to identify the responsible structures and to characterize the adsorptive mechanism.

Although cholesterol lowering effects are described for both lupin and maize derived dietary fibre enriched ingredients, there is a lack of in vivo data that directly correlates this effect with bile acid interactions [70,71]. On the one hand, this could be explained by the low overall number of studies regarding these specific fibres. On the other hand, it could be concluded that interaction with bile acids are not the main mechanism of cholesterol reduction. While the maize fibre preparation did not significantly increase the viscosity of the digesta, the viscosity of chyme containing lupin fibre was comparable with the fibre preparations derived from pea and potato. Concomitantly, there are no in vivo studies on these fibres indicating positive nutritional effects due to increased viscosities. Therefore, potential viscosity effects of this magnitude are either insufficient to cause significant outcomes or are superimposed on other effects during in vivo digestion. To the best of our knowledge there are no former studies describing the adsorptive effects of lupin and maize fibre preparations on CDCA and DCA. The enhanced binding of DCA might be a possible explanation for the increased excretion and accumulation of primary bile acids in the bile acid pool already described in in vivo studies using blue lupin products [70,72].

Responsible structures and mechanisms of bile acid retention remain to be fully elucidated. To understand structure function relationships detailed knowledge of the plant tissue, processing of fibre preparations and their physiochemical properties is required. Future in vitro studies focusing on bile acid retention should be combined with dietary fibre characterisations. Furthermore, to understand the relationship of dietary fibre structures and health, characterization and reporting of dietary fibre sources is crucially needed in in vivo studies to identify responsible compounds.

## 5. Conclusions

The comparability of our results for fibre enriched food ingredients showing different structural and functional properties with in vivo studies, especially with ileostomy studies, highlights the advantages of the applied in vitro method to study interactions with bile acids. The influence of micelle formation on the bile acid release demonstrated further illustrates the limitation of many studies using concentrations below the CMC [10]. We were able to link viscosity and adsorption as influenced by fibre sources with the structural properties of the main bile acids abundant in human bile. Adsorption directly correlated to the hydrophobicity of the bile acids, which supports the hypothesis of a hydrophobic linkage between bile acids and dietary fibre. Delayed diffusion in viscous fibre matrices was further associated with the micellar properties of the bile acids. Secondary bile acids, especially DCA, are associated with a number of disease phenotypes [7]. DCA is known to accumulate in the bile acid pool, if a “Western diet” low in fibre is consumed. Our results suggest that the extent of the interaction of DCA with bile acids is increased by its hydrophobicity and micellar properties. The elucidation of this increased interaction in comparison to primary bile acids is especially important as the human liver is unable to undertake 7α-hydroxylation of secondary bile acids [4]. Our results could therefore contribute to the elucidation of mechanisms responsible for the health promoting effects of dietary fibres. The applied in vitro methodology could act as an initial indicator to identify principal mechanisms and structures responsible for bile acid retention. However, dialysis is a simplified model to simulate absorption. The influence of the mucous layer, small intestinal peristaltic and active bile acid transporters are not covered by this model. To ensure the transferability to physiological processes, in vivo studies need to be conducted to corroborate the presented in vitro findings. As dietary fibre intake and changes in bile acid profiles are directly related to microbial shifts and the activity of the gut microbiota [73,74], future studies need to focus on the interplay between dietary fibre, bile acids and the microbiome.

## Figures and Tables

**Table 1:** Dietary fibre composition (soluble dietary fibre (SDF), insoluble fibre (IDF), and total dietary fibre (TDF), *n* = 3) of dietary fibre enriched food ingredients derived from different sources.

Source	SDF	IDF	TDF	SDF/TDF
	[g/100 g DM]	[g/100 g DM]	[g/100 g DM]	[%]
apple	15.7 ± 1.6	73.0 ± 1.9	88.7 ± 2.5	17.6 ± 1.3
barley ^1^	19.8 ± 1.5	9.5 ± 0.2	29.2 ± 1.5	67.3 ± 1.0
citrus ^1^	14.9 ± 0.3	79.8 ± 1.2	94.7 ± 1.2	15.7 ± 0.3
lupin ^1^	4.8 ± 0.6	78.6 ± 0.4	83.4 ± 0.7	5.8 ± 0.7
maize	1.3 ± 0.8	78.9 ± 0.8	80.2 ± 1.1	1.6 ± 1.0
oat	1.4 ± 1.1	92.6 ± 0.4	94.0 ± 1.2	1.5 ± 1.1
pea	2.0 ± 0.4	52.8 ± 0.1	54.8 ± 0.5	3.6 ± 0.8
potato ^1^	11.2 ± 1.7	56.5 ± 0.8	67.8 ± 1.9	16.5 ± 2.2
res. starch ^2^	84.7 ± 0.7	-	-	-
wheat	2.5 ± 0.2	96.0 ± 0.1	98.5 ± 0.2	2.5 ± 0.2

^1^ as published by Naumann et al. (2018) determined by AOAC 991.43 [17]; ^2^ as published by Lefranc-Millot et al. (2010) determined by AOAC 2001.03 [26]; - not detected.

**Table 2:** Bile acid adsorption of dietary fibre enriched food ingredients (given as sum of glyco- and tauroconjugated cholic acids (CA), chenodesoxycholic acids (CDCA), desoxycholic acids (DCA)).

Source	bile acid adsorption [μmol/100 mg DM]		
	CA	CDCA	DCA
apple	0.11 ± 0.05 ^a^	0.56 ± 0.18 ^a^	0.29 ± 0.97 ^a^
barley	1.19 ± 0.13 ^b^	3.20 ± 0.32 ^c^	4.65 ± 0.17 ^d^
citrus	0.38 ± 0.48 ^a^	0.37 ± 0.42 ^a^	0.25 ± 0.14 ^a^
lupin	0.44 ± 0.15 ^a^	1.74 ± 0.26 ^b^	2.18 ± 0.11 ^c^
maize	0.38 ± 0.13 ^a^	1.87 ± 0.10 ^b^	2.88 ± 0.24 ^c^
oat	1.07 ± 0.11 ^b^	2.57 ± 0.06 ^c^	2.01 ± 0.57 ^c^
pea	−0.04 ± 0.21 ^a^	0.65 ± 0.28 ^a^	0.87 ± 0.16 ^b^
potato	−0.10 ± 0.29 ^a^	0.29 ± 0.29 ^a^	0.72 ± 0.28 ^a^
res. starch	0.03 ± 0.05 ^a^	0.21 ± 0.08 ^a^	0.19 ± 0.07 ^a^
wheat	0.09 ± 0.11 ^a^	0.18 ± 0.12 ^a^	−0.03 ± 0.28 ^a^
blank	0.09 ± 0.01 ^a^	0.02 ± 0.11 ^a^	0.01 ± 0.19 ^a^

Along the column, different letters indicate significant differences on a *p* ≤ 0.05 level basis.

**Table 3:** Apparent permeability rate constants (k) of kinetic bile acids release analysis (glycocholic acid (GCA), taurocholic acid (TCA), glycochenodesoxycholic acid (GCDCA), taurochenodesoxycholic acid (TCDCA), glycodesoxycholic acid (GDCA), taurodesoxycholic acid (TDCA)) of dietary fibre enriched food ingredients derived from different sources.

	Apparent Permeability Rate k (h^−1^)					
Source	GCA	TCA	GCDCA	TCDCA	GDCA	TDCA
apple	0.26 ± 0.01 ^a^	0.27 ± 0.01 ^a^	0.18 ± 0.01 ^a^	0.17 ± 0.01 ^a^	0.12 ± 0.02 ^a^	0.12 ± 0.02 ^a^
barley	0.35 ± 0.04 ^a,b^	0.31 ± 0.04 ^a^	0.25 ± 0.01 ^a^	0.24 ± 0.01 ^a,b^	0.30 ± 0.05 ^b,c^	0.28 ± 0.02 ^b,c^
citrus	0.26 ± 0.06 ^a^	0.27 ± 0.04 ^a^	0.17 ± 0.04 ^a,b^	0.17 ± 0.04 ^a^	0.15 ± 0.01 ^a,b^	0.15 ± 0.01 ^a,b^
lupin	0.26 ± 0.06 ^a^	0.46 ± 0.04 ^a^	0.39 ± 0.01 ^b,c^	0.34 ± 0.02 ^b,c^	0.36 ± 0.07 ^c,d^	0.34 ± 0.04 ^c,d^
maize	0.54 ± 0.05 ^b^	0.86 ± 0.10 ^b^	0.46 ± 0.05 ^c,d^	0.45 ± 0.05 ^c,d^	0.33 ± 0.04 ^c,d^	0.33 ± 0.03 ^c^
oat	0.85 ± 0.12 ^c^	0.74 ± 0.12 ^b^	0.55 ± 0.11 ^d,e,f^	0.52 ± 0.11 ^d,e,f^	0.54 ± 0.04 ^e,f^	0.53 ± 0.04 ^f,g^
pea	0.79 ± 0.17 ^c^	0.77 ± 0.03 ^b^	0.51 ± 0.04 ^c,d,e^	0.55 ± 0.08 ^d,e,f^	0.39 ± 0.07 ^c,d,e^	0.37 ± 0.05 ^c,d,e^
potato	0.88 ± 0.06 ^c^	0.89 ± 0.07 ^b^	0.55 ± 0.04 ^d,e,f^	0.52 ± 0.07 ^d,e^	0.39 ± 0.11 ^c,d,e^	0.38 ± 0.10 ^c,d,e,f^
res. starch	0.92 ± 0.07 ^c^	0.85 ± 0.08 ^b^	0.63 ± 0.04 ^e,f,g^	0.60 ± 0.04 ^d,e,f^	0.50 ± 0.05 ^d,e,,f^	0.48 ± 0.04 ^d,e,f^
wheat	0.92 ± 0.10 ^c^	0.80 ± 0.09 ^b^	0.68 ± 0.08 ^f,g^	0.63 ± 0.07 ^e,f^	0.53 ± 0.03 ^e,f^	0.51 ± 0.04 ^e,f,g^
blank	1.02 ± 0.07 ^c^	0.90 ± 0.15 ^b^	0.74 ± 0.05 ^g^	0.68 ± 0.04 ^f^	0.61 ± 0.09 ^f^	0.58 ± 0.08 ^g^

Along the column, different letters indicate significant differences on a *p* ≤ 0.05 level basis.

**Table 4:** Critical micelle concentration (CMC) and aggregation number (N_agg_) of main bile acids as summarized by Parker et al. taken from Madenci and Egelhaaf [37,38].

Bile Acid	Abbreviation	CMC [mM]	N_agg_
taurocholic acid	TCA	3–18	3–7
glycocholic acid	GCA	4	9
taurochenodesoxycholic acid	TCDCA	0.9–7	5–26
glycochenodesoxycholic acid	GCDCA	1–2	15
taurodesxycholic acid	TDCA	2–3	12–19
glycodesoxycholic acid	GDCA	1–2	13–16

## Abbreviations

CA	cholic acid
CDCA	chenodesoxycholic acid
CMC	critical micelle concentration
DCA	desoxycholic acid
DM	dry matter
GCA	glycocholic acid
GCDCA	glycochenodesoxycholic acid
GDCA	glycodesoxycholic acid
RS	resistant starch
TCA	taurocholic acid
TCDCA	taurochenodesoxycholic acid
TDCA	taurodesoxycholic acid
